# *PCM1-JAK2* Fusion Tyrosine Kinase Gene-Related Neoplasia: A Systematic Review of the Clinical Literature

**DOI:** 10.1093/oncolo/oyac072

**Published:** 2022-04-26

**Authors:** Henry G Kaplan, Ruyun Jin, Carlo B Bifulco, James M Scanlan, David R Corwin

**Affiliations:** Swedish Cancer Institute, Seattle, WA, USA; Center for Cardiovascular Analytics, Research and Data Science (CARDS), Providence Heart Institute, Providence Research Network, Portland, OR, USA; Providence St Joseph Health System, Portland, OR, USA; Swedish Center for Research and Innovation, Seattle, WA, USA; CellNetix, Seattle, WA, USA; Swedish Medical Center, Seattle, WA, USA

**Keywords:** *PCM1-JAK2*, eosinophilia, myelodysplastic neoplasia, leukemia

## Abstract

**Background:**

This review summarizes the case studies of *PCM1-JAK2* fusion tyrosine kinase gene-related neoplasia. Recommended treatment includes *JAK2* inhibitors and hematologic stem cell transplantation (HSCT), although the small number of patients has limited study of their efficacy. Herein, we present all available cases in the current searchable literature with their demographics, diagnoses, treatments, and outcomes.

**Methods:**

PubMed, ScienceDirect, Publons, the Cochrane Library, and Google were searched with the following terms: *PCM1-JAK2*, ruxolitinib and myeloid/lymphoid.

**Results:**

Sixty-six patients (mean age = 50, 77% male) had an initial diagnosis of myeloproliferative neoplasm (MPN) in 40, acute leukemia in 21 and T-cell cutaneous lymphoma in 5. Thirty-five patients (53%) had completed 5-year follow-up. The 5-year survival for the MPN, acute myelogenous leukemia (AML), acute lymphocytic leukemia, and lymphoma groups are 62.7, 14.9%, 40.0%, and 100%, respectively. Too few patients have been treated with ruxolitinib to draw conclusions regarding its effect on survival while the 5-year survival for MPN patients with or without HSCT was 80.2% (40.3%-94.8%) versus 51.5% (22.3%-74.6%), respectively. The T-cell cutaneous lymphoma patients have all survived at least 7 years.

**Conclusion:**

This rare condition may be increasingly detected with wider use of genomics. Ruxolitinib can yield hematologic and molecular remissions. However, HSCT is, at this time, the only potentially curative treatment. Useful prognostic markers are needed to determine appropriate timing for HSCT in patients with MPN. Patients presenting with acute leukemia have a poor prognosis.

## Teaser

This review summarizes the literature on case studies of *PCM1-JAK2* fusion tyrosine kinase gene-related neoplasia, focusing on the clinical course of the disease and the efficacy of treatments used to date.

**Implications for Practice**

While *PCM1-JAK2* fusion is an uncommon condition it affects a wide variety of hematopoietic cell lines and therefore can present as a myeloproliferative neoplasm (MPN), acute leukemia, non-Hodgkin lymphoma (including mycosis fungoides), and perhaps Hodgkin disease. Eosinophilia and erythrocyte dysplasia can be prominent clues to the diagnosis. While cytogenetics often can demonstrate the fusion, specific probes, and next generation sequencing may be required. *JAK2* inhibitors may be helpful in MPN and stem cell transplantation has been successful in a number of cases. Clarifying indicators for early transplantation would be helpful. Patients presenting with acute leukemia have a very poor prognosis.

## Introduction

A variety of *JAK2* variants that activate tyrosine kinases have been reported, including the well-known *V617F* variant which occurs in most patients with polycythemia vera and approximately half of the patients with essential thrombocytosis and primary myelofibrosis.^1–5^ Many of these patients will respond favorably to *JAK2* inhibitors.^6,7^ Translocations and rearrangements involving *JAK2*, however, are less common. These variants affect many hematopoietic cell lines and may lead to malignancy in any of them.^8–10^ They are now classified by the World Health Organization as “Myeloid/lymphoid Neoplasms with Eosinophilia and TK Fusion Genes.”^8–15^ While the diagnosis can usually be made with routine cytogenetic studies, it can sometimes require specific probes, FISH, and next generation sequencing.^16-19^ This is extremely important as different therapies may be indicated for different variants. For example, within this broader group of neoplasms, fusions of *PDGFRA* and *PDGFRB* with a variety of gene partners (not including *JAK2*) have been reported to respond well to imatinib.^20–24^ The general approach to these fusion variants is discussed in the current National Comprehensive Cancer Network (NCCN) guidelines.^8^

In this report, we take the opportunity to review the cases reported in the literature specifically with the *PCM1-JAK2* variant to assess what is known about the clinical course of this disease and the efficacy of treatments that have been used to this point in time.

## Review of the Clinical Literature

**Figure 1.**
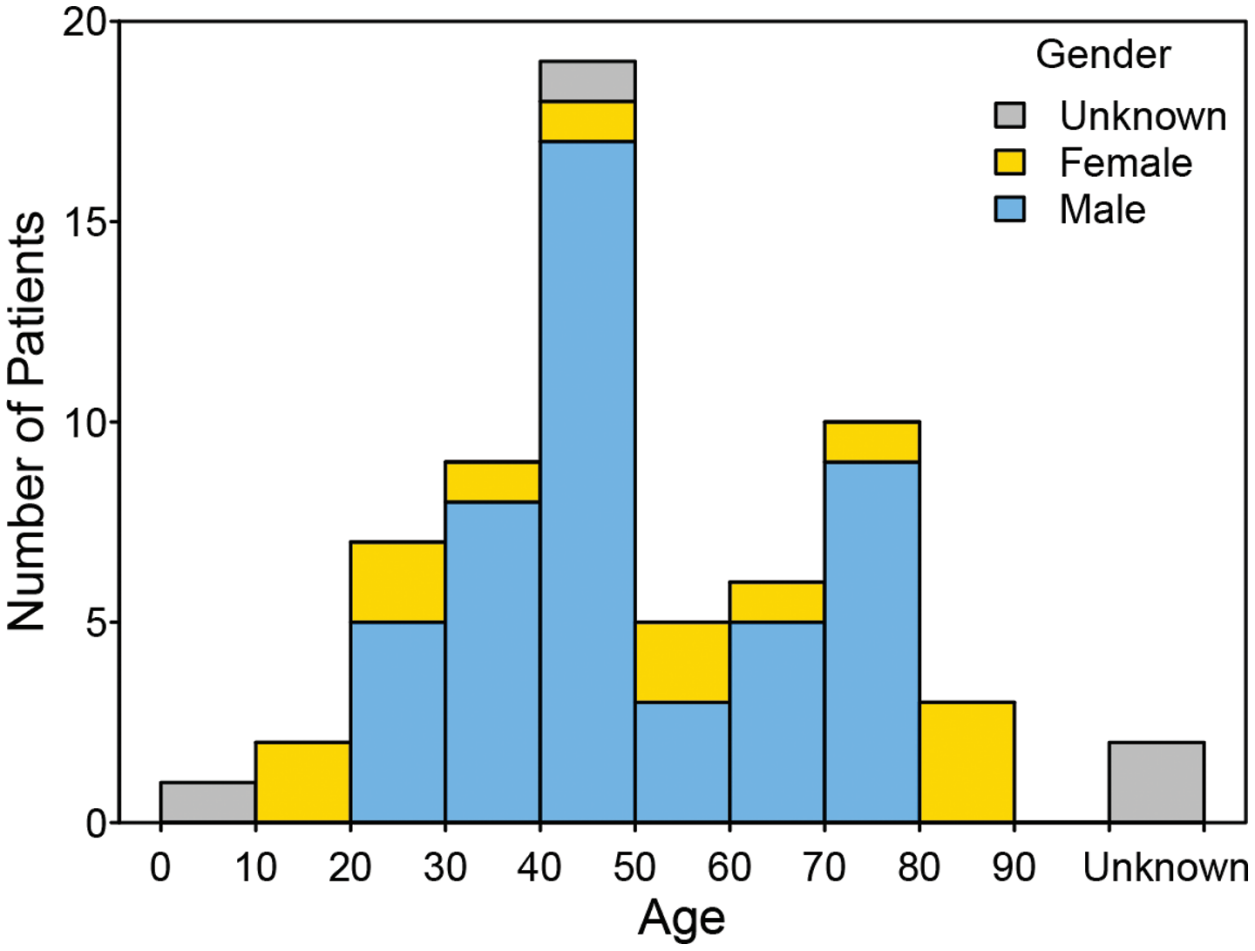
Age and gender distribution in 64 patients with *PCM1-JAK2*.

In our review of the literature as of September 5, 2021 interrogating PubMed, Science Direct, Publons, Cochrane Library, and Google utilizing search terms *PCM1-JAK2*, ruxolitinib, and myeloid/lymphoid we were able to identify 66 cases harboring the *PCM1-JAK2* fusion mutation. These patients spanned the entire gamut of ages with a median (interquartile range) of 47 (36.5, 69.3) and range of 6 to 86 years (Fig. 1 and Table 1). Forty-eight of the 62 (77%) that reported patient gender were male. The reason for the male preponderance is not clear. Some of these reports overlap with the same patients included in multiple studies, though this overlap has been eliminated in this review by curation of the individual case reports.

**Table 1.** Clinical course of patients with *PCM1-JAK2* fusion variant.

ID	Author	Initial Dx	Eosinophilia	Initial Rx	Duration 1st phase (months)	Transformed Dx	2nd Rx	Survival after 2nd Rx in months	Age	Sex	Survival in months
1	Bousquet^25^	MPN	No	HU, HSCT	7			4	46	M	12
2	Reiter^26^	MPN	No	None	10	ALL	HSCT	53+	32	M	63+
3	Reiter^26^	MPN	Yes	IFN, then HSCT	10			13+	42	M	23+
4	Reiter^26^	MPN	Yes	None	72	AML			74	M	73
5	Schwaab^27^	MPN	No	Ruxolitininb—CHR					70	M	26+
6	Cornfield^28^	MPN	Yes	Imatinib, HU several month, then HSCT					45	M	28+
7	Dargent^29^	MPN	Yes	HU	30+				57	M	30+
8	Lierman^30^	MPN	Yes	HU	15		Ruxolitinib, PR	15+	72	M	30+
9	Precup^31^	MPN	Yes	IFN + prednisone, PR	12+	Myelofibrosis			47		12+
10	Prochorec- Sobieszek^32^	MPN	Yes	IFN, HU, Ara C	30		HSCT	3+	22	F	33+
11	Reiter^26^	MPN	Yes	IFN—CR					47	M	89
12	Rumi^33^	MPN	Yes	Ruxolitinib, CR	46+				31	F	46+
13	Rumi^33^	MPN		Ruxolitinib	36+				72	M	39
14	Murati^34^	MPN	Yes	HU + IFN, HSCT				60+	43	M	60+
15	Reiter^26^	MPN		None					72	M	0.1
16	Schwaab^27^	MPN	Yes	Ruxolitinib + HSCT (preplanned)	2	HD after HSCT		43+	29	M	45+
17	Schwaab^27^	MPN	No	Ruxolitinib—no response	1				76	M	4
18	Tang^35^	MPN	No	HU		AML			43	M	35
19	Heiss^36^	MPN	Yes	HU	10	Acute erythroleukemia	HSCT		61	M	10+
20	Kaplan^37^	MPN	No	None	27	T-cell ALL	HyperCVAD, HSCT	41+	28	F	68+
21	Schwaab^27^	MPN		Ruxolitininb—CHR	38	Progressive disease	HSCT	5+	49	M	43+
22	Schwaab^27^	MPN	Yes	RUXOLITINIB—CHR	26	Clonal evolution; Burkitt after HSCT	HSCT	5	50	M	31
23	Schwaab^27^	MPN	Yes	Ruxolitininb—CHR	32	Progressive disease	HSCT	37+	51	M	69+
24	Chase^38^	MPN									
25	Murati^34^	MPN	Yes	HU 2 months, IFN + HU + Ara C			HU + IFN + Ara C	20	30	M	22
26	Murati^34^	MPN	Yes	HU 1 month, IFN 1 year, imatinib 9 months	21	AML			45	M	21+
27	Patterer^39^	MPN	Yes	Ruxolitinib, splenectomy					50	M	16+
28	Patterer^39^	MPN	Yes	HU, Ara C					72	M	5
29	Saba^40^	MPN							35	M	
30	Stewart^36^	MPN	Yes	HU, splenectomy					24	M	12
31	Tang^35^	MPN		Decitabine					82	F	8+
32	Tang^35^	MPN	No	HU, ATRA					86	F	1
33	Song^41^	MPN	No	After 13 months DNM+Ara C	13+			13+	42	M	13+
34	Tang^35^	MPN	Yes	9-Aminocamptotehecin					37	M	24+
35	Tang^35^	MPN	Yes	Ruxolitinib, HSCT					40	M	29+
36	Tang^35^	MPN	Yes	HU					53	F	104+
37	Tang^35^	MPN	Yes	None					70	M	13+
38	Tang^35^	MPN	Yes	None					71	M	142
39	Dunlap^42^	MPN									
40	Patnaik^43^	MPN							78	M	
41	Bousquet^25^	AML	Yes	Ara C + ida + XRT, CR					44	M	1+
42	Huang^44^	AML		Ara C + ida + Hi Ara C—CR			Fludarabine, Ara C	1	48	F	7
43	Luedke^45^	AML	No						32	M	
44	Masselli^46^	AML		Induction chemo + HSCT	3.3+				29	M	3.3+
45	Patterer^39^	AML	Yes	Ara C + doxorubicin					47	M	6
46	Patterer^39^	AML		palliative care					73	M	0.3
47	Patterer^39^	AML		palliative care					75	F	7+
48	Reiter^26^	AML		induction-CR, then IFN 6 yrs					54	M	180+
49	Schwaab^27^	AML	No	Ruxolitinib—no response	2		Azacytidine	12	69	F	14
50	Salehi^47^	APL	Yes	Arsenic Trioxide, all trans-retinoic acid					86	F	1
51	Cheng^48^	Erythroleukemia		Ruxolitinib, HSCT					40	M	2+
52	Lee^49^	Erythroleukemia	No	Induction chemo-CR 19 mths; reinduction, HSCT					51	F	29
53	Murati^34^	Erythroleukemia	No	CDDP, VP16, ifos			LAME91 protocol		12	F	10
54	Patterer^39^	B-cell ALL		GMALL protocol, clin CR, genomic PR	4				50	M	8
55	Tang^35^	B-cell ALL	No	HyperCVAD					47	M	2
56	Tang^35^	B-cell ALL	No	HyperCVAD, inotuzumab, rituxan					69	M	7+
57	Wouters^50^	B-cell ALL	No	VCR, DNM, pred, IT MTX;Ara C, 6-TG, VP-16	12		Ruxolitinib	12+	77	F	24+
58	Reiter^26^	Pre-B-cell ALL		Chemo					50	M	1
59	Schwaab^27^	Pre-B-cell ALL	No	Induction chemo + HSCT		Progressive disease	Ruxolitinib	6	63	M	6
60	Adelaide^51^	T-cell ALL		VAC + consolidation—CR	72	Relapse	VAC + Lasp + Hi Ara C/Hi MTX-CR, HSCT	7+	40	M	85+
61	Tsai^52^	T-cell ALL		Induction chemo	2	AML	I3A7 induction + Hi Ara C		43	M	2+
62	Fernandez-Pol^53^	Mycosis fungoides	No	Systemic Therapy + Radiation					14	F	132+
63	Fernandez-Pol^53^	Mycosis fungoides	No	Brentuximab					30	M	84+
64	Fitzpatrick^54^	Peripheral T-cell lymphoma							28	M	
65	Davis^55^	T-cell lymphomatoid papulosis/ mycosis fungoides		XRT	~48	“HD”	XRT-CR	~144, then T-cell lymphoma	31	M	~204
66	Riedlinger^56^	T-cell lymphoma/mycosis fungoides		UV therapy + psoralen	~144	“HD”, T-cell lymphoma	BEACOPP		6		204+

PTCL, peripheral T-cell lymphoma; IFN, interferon; HSCT, hematologic stem cell transplantation; Ara C, cytosine arabinoside; DNM, daunomycin; Ida, idarubicin; CDDP, cisplatin; VP-16, etoposide; ifos, ifosphamide; IT MTX, intrathecal methotrexate; 6-TG, 6-thioguanine; pred, prednisone; blinat, blinatumumab; CR, complete remission; CHR, complete hematologic remission.

**Figure 2.**
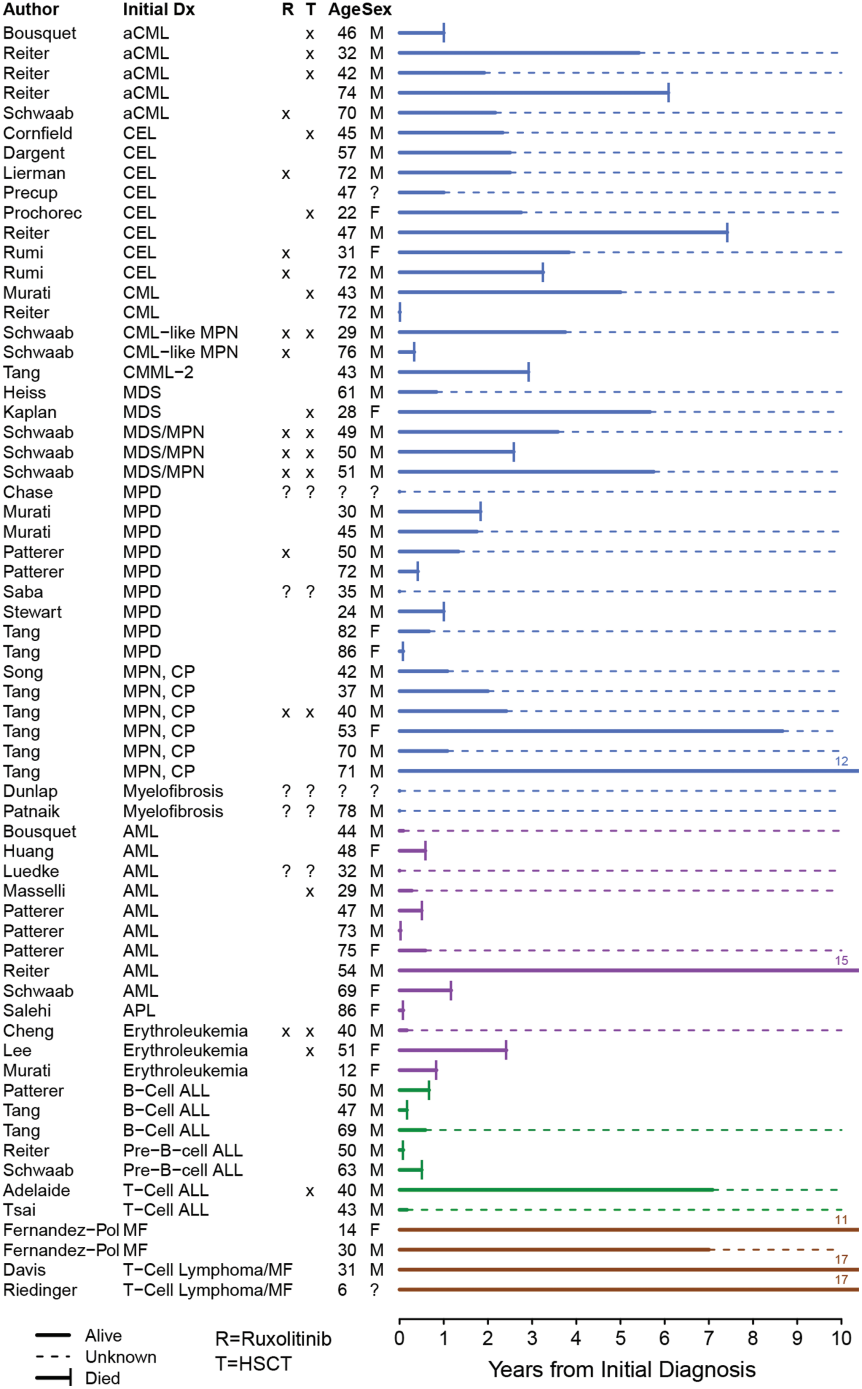
Swimmer plot showing survival and treatments of the 64 *PCM1-JAK2* patients by the initial diagnosis. Abbreviations: aCML, atypical chronic myelogenous leukemia; ALL, acute lymphocytic leukemia; AML, acute myelogenous leukemia; APL, acute promyelocytic leukemia; CEL, chronic eosinophilic leukemia; CML, chronic myeloid leukemia; CMML, chronic myelomonocytic leukemia; CP, chronic phase; F, female; HSCT, hematologic stem cell transplant; M, male; MDS, myelodysplastic syndrome; MF, mycosis fungoides; MPD, myeloproliferative disease; MPN, myeloproliferative neoplasm.

In assessing all 66 patients, 40 carried a variety of initial diagnoses that we have curated to be consistent with a myeloproliferative neoplasm (MPN; Fig. 2 and Table 1). These cases included atypical chronic myelogenous leukemia and chronic eosinophilic leukemia. In Fig. 2, we have retained the original diagnoses noted in the source manuscripts to provide historical perspective. However, per current terminology, they are noted as MPN’s in other figures and Table 1. There were also 21 cases of acute leukemia at first diagnosis (32%), including 9 cases of acute myelogenous leukemia (AML), 3 cases of the AML subgroup acute erythroleukemia (AEL) and 1 of acute promyelocytic leukemia (APL). There were 6 cases of B-cell acute lymphoblastic leukemia (ALL), and 2 of T-cell ALL. There were also 5 lymphoma patients. In this report we have summarized the individual cases (Table 1) and discuss the course of disease by diagnostic category. Six reports lack clinical outcome data, though they are included in Table 1 and Fig. 2.^38,40,42,43,45,54^

## Five-Year Survival by Initial Diagnosis

Long-term survivals were analyzed using the Kaplan-Meier estimate and compared by the log-rank test. Median survival time was reported with the 95% confidence interval (CI). Statistical analyses were performed using R version 4.0.3 (R Core Team (2020). R: A language and environment for statistical computing. R Foundation for Statistical Computing, Vienna, Austria. https://www.R-project.org).

**Figure 3.**
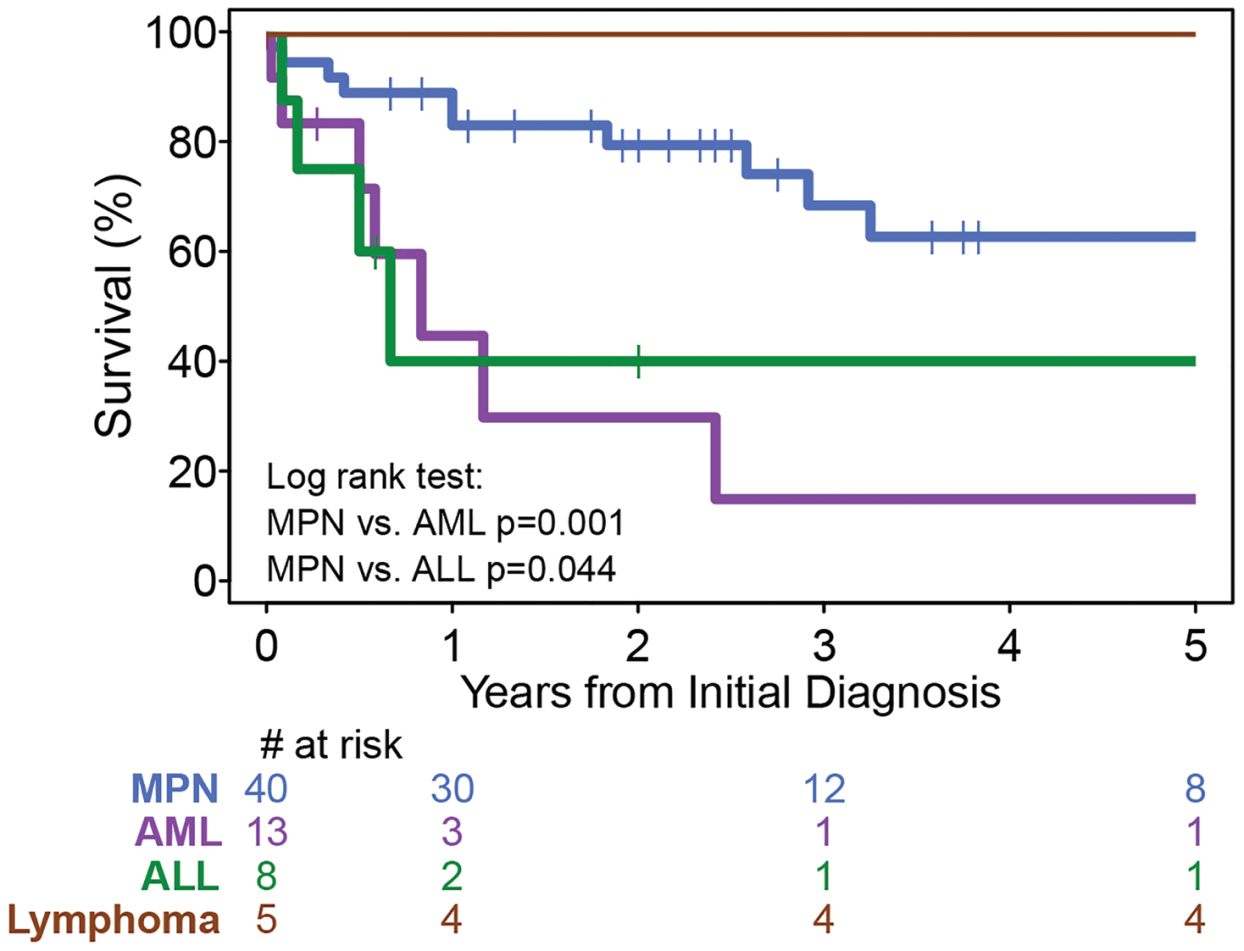
Kaplan-Meier plot of survival by initial diagnosis related to *PCM1-JAK2*. Abbreviations: AML, acute myelogenous leukemia; ALL, acute lymphocytic leukemia; MPN, myeloproliferative neoplasm.

Based on the reported data in the literature, only 35 patients (53%) had completed 5-year follow-up (45%, 62%, 63%, and 80% in the MPN, AML, ALL, and Lymphoma initial diagnosis groups, respectively). The survival by the above 4 groups are shown in Fig. 3. The median survival time for AML and ALL groups are 10 and 8 months, respectively. The 5-year survival for the MPN, AML, ALL, and lymphoma initial diagnosis groups are 62.7% (95% CI 39.6-79.0%), 14.9% (0.8-47.3%), 40.0% (6.6-73.4%), and 100%, respectively.

## Myeloproliferative Neoplasms

The *PCM1-JAK2* fusion was first described as a syndrome of MPNs in multiple patients with acute and chronic leukemia by Reiter in 2005.^26^ Rearrangements have been noted to involve a variety of breakpoints in both genes. All of the fusions contain a number of the coiled-coil domains of *PCM1* and the complete catalytic tyrosine kinase domain of *JAK2.*^57^ This leads to the constitutive activation of the *JAK2* kinase due to the oligomerization mediated by the coiled-coil domains of *PCM1*, which in turn, activates the JAK/STAT axis. All of these fusion rearrangements have been associated with eosinophilia and not surprisingly, in view of the polycythemia noted with the *V617F* mutation, dysplastic erythroid proliferation has been prominently seen as well.^32^

**Figure 4.**
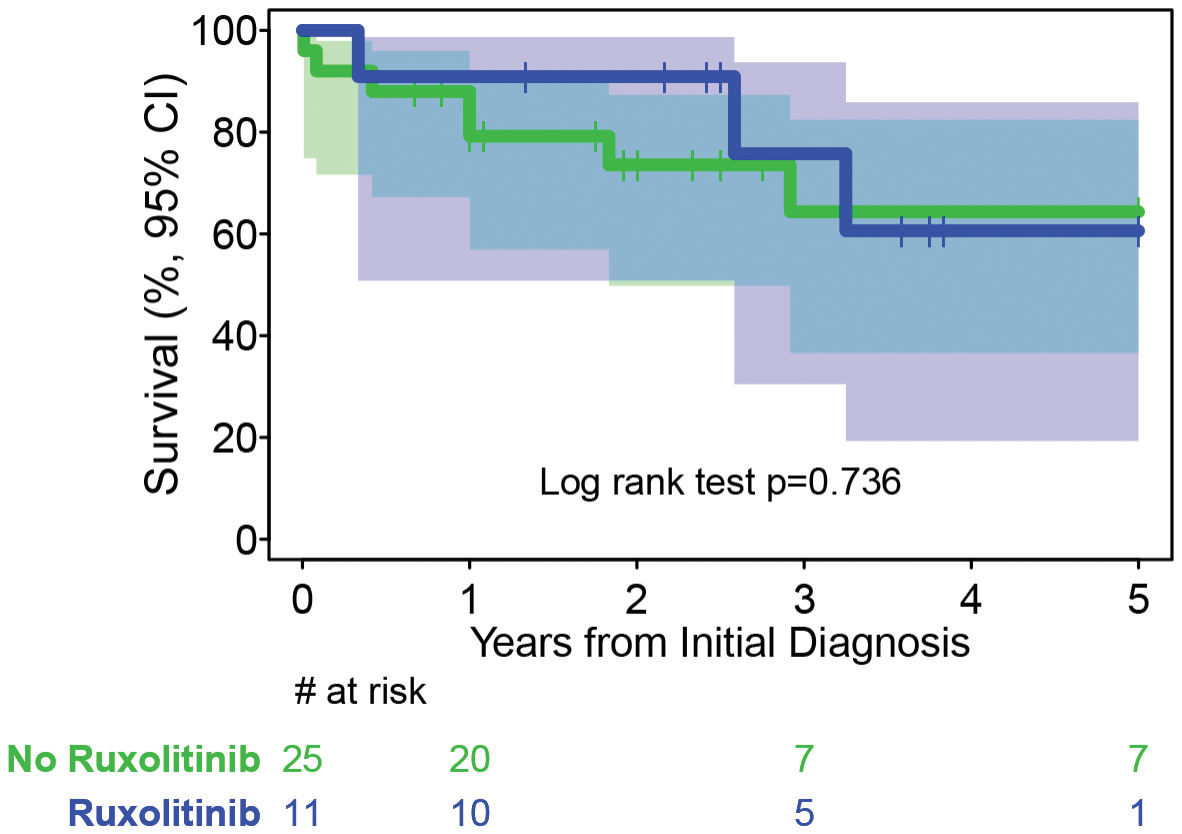
Kaplan-Meier survival curves of patients with MPN with or without ruxolitinib.

**Figure 5.**
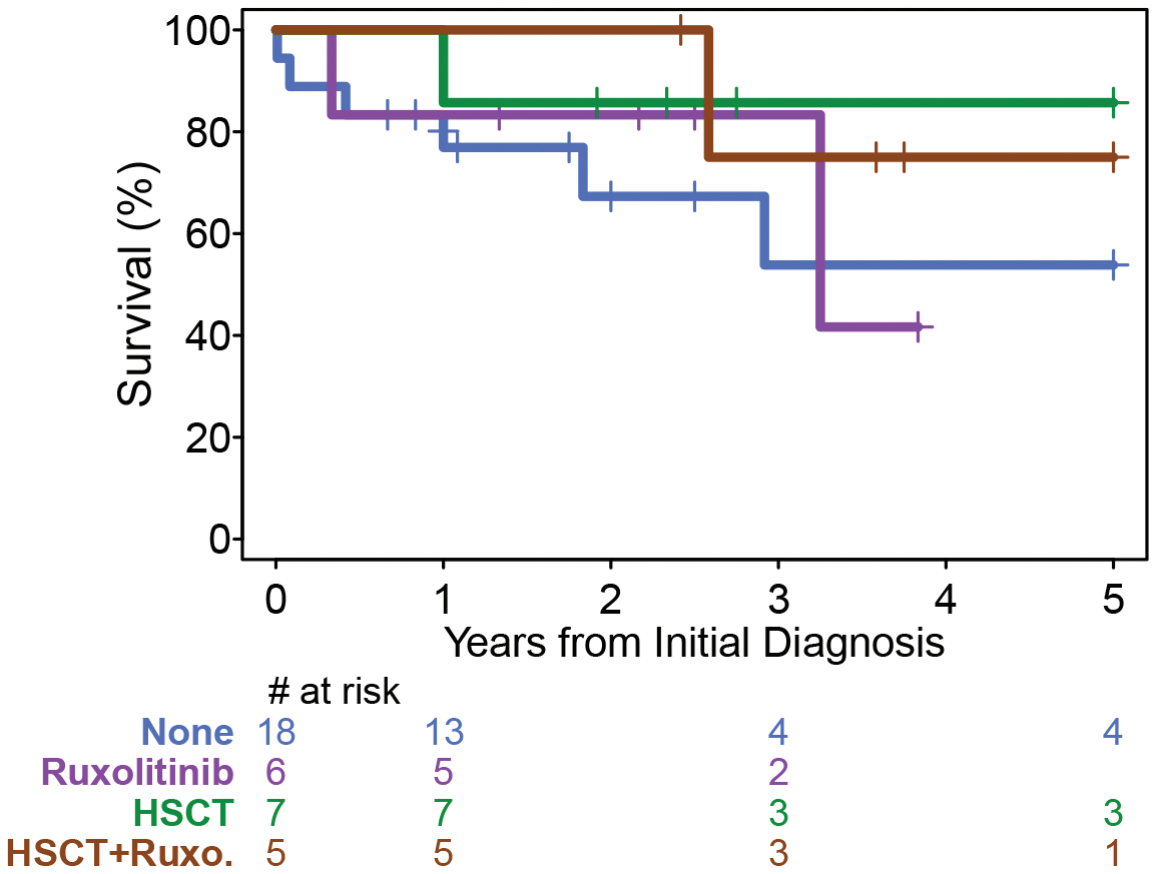
Kaplan-Meier survival curves of patients with MPN with or without ruxolitinib and stem cell transplant.

Ruxolitinib is a potent *JAK2* inhibitor that is widely used to treat polycythemia vera and myelofibrosis.^6,7^ Studies have evaluated this drug in cell lines with a variety of *JAK2* fusion variants, including *PCM1-JAK2,* and found it to be active at nanomolar concentrations.^11,12,33,38^ Ruxolitinib has produced significant clinical responses in this type of MPN, including hematologic remissions. Eleven patients in this series received ruxolitinib for MPN.^27,30,33,35,39^ The 5-year survival for MPN patients treated with or without ruxolitinib were 60.6% (95% CI 19.3%-85.9%) versus 64.4% (95% CI 36.6%-82.4%), respectively, *P* = .736 (Fig. 4). The median survival for patients not treated with ruxolitinib was 89 months and not enough data to compute for ruxolitinib-treated patients. However, this may be biased by the fact that the ruxolitinib group is a smaller, more recent group than those who did not receive this drug.. Analysis of ruxolitinib’s effect on survival is also complicated by the fact that 5 of these patients also received HSCT.^27,30,33,39^ For these 5 patients^,27,35^ the 5-year survival was 75.0% (95% CI 12.8%-96.1%) and for the 6 who did not receive transplant^27,30,33,39^ the 5-year survival was unavailable (Fig. 5). For the 25 MPN patients who did not receive ruxolitinib, 7 received HSCT^25,28,32,34,36,37^ and had 5-year survival of 85.7% (95% CI 33.4%-97.9%); 18 patients who did not receive HSCT^26,29,31,34-36,39,41,58^ had 5-year survival of 53.8% (95% CI 21.0%-78.2%; Fig. 5).

**Figure 6.**
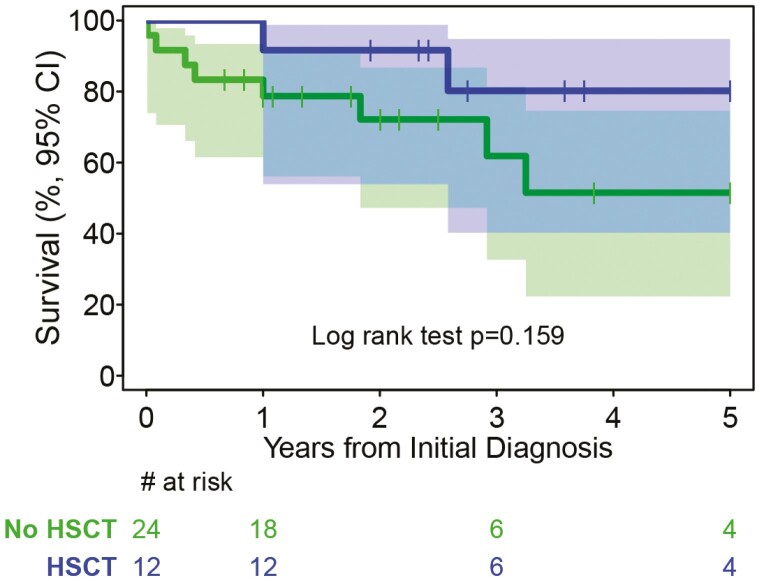
Kaplan-Meier survival curves of patients with MPN with or without stem cell transplant.

Considering the entire cohort of 36 MPN patients for whom we have clinical data, the 5-year survival for MPN patients with or without HSCT was 80.2% (95% CI 40.3%-94.8%) versus 51.5% (95% CI 22.3%-74.6%), respectively, *P* = .159 (Fig. 6). The median survival for no HSCT patients was 73 months and not enough data to compute for HSCT patients (Fig. 6).

Seven patients underwent HSCT while still in chronic phases of MPN.^,25-27,32,34,35^ Three other patients were transplanted in a state of “progressive disease” or “clonal evolution,” though they were not reported to have actually transformed into acute leukemia.^27^ Eight of these patients were still alive at the time of reportage (Table 1).

A variety of other therapies have been undertaken in chronic phase MPN either simultaneously or sequentially, 14 patients have received hydroxyurea, 5 received interferon, 4 received cytosine arabinoside, 2 received imatinib and 1 each of daunomycin, 9-aminocamptothecan, and ATRA have been noted. Six patients received no therapy whatsoever for chronic phase MPN, with 3 transforming to acute leukemia at 10, 27, and 72 months,^26,37^ while 3 others did not transform and survived 0.1, 13+, and 142 months.^26,35^

The NCCN guidelines recommend *JAK2* inhibitors or experimental therapy with consideration of early transplant for these MPN patients.^8^ With data reported for only 11 patients treated with ruxolitinib for MPN it is not possible to draw any conclusions regarding its effect on survival. Schwaab has suggested that the role of ruxolitinib be as a temporizing measure prior to transplantation.^27^ It will be important to evaluate prognostic factors that might predict early transformation to guide timing of transplantation, given the presence of untreated and ruxolitinib only treated survivors ranging beyond 5 years.

## Acute Leukemia

Among the *PCM1-JAK2* patients presenting with acute leukemia, survivals were short^25,27,34,39,44,47^ with the exception of a patient with AML with prolonged interferon therapy alive at 180 months^26^ and the T-cell patient with ALL alive at 85+ months.^51^ One patient with ALL was transplanted and was alive at 3+ months^46^ and one patient was alive at 2+ months immediately after HSCT.^48^ The patient reported by Lee did not obtain remission with HSCT and survived 29 months.^49^ Uniquely, the patient reported by Tsai quickly transformed from ALL to AML but there is no follow-up beyond that point.^52^ A single response to ruxolitinib treatment for acute leukemia was recently reported by Wouters in an elderly woman after chemotherapy for B-cell ALL who had an ongoing hematologic but not molecular complete response to ruxolitinib at 12 months.^50^ One patient with AML has been treated with ruxolitinib with no response.^27^

Six patients with MPN transformed into acute leukemia. Two developed ALL (one B cell^54^ and one T cell^37^) and 4 AML^26,34,35^ (including one erythroleukemia^36^). Both patients with ALL received HSCT and are alive at 68+ and 63+ months (41+ and 53+ months after transplant).^26,37^ The patients’ with AML overall survivals were 10+, 21+, 35, and 73 months but survival after transformation is either short or not reported.

It should be noted that 2 cases which are included in the acute leukemia group were felt by the original treating physicians to have likely evolved from MPN though they presented as acute leukemia.^26,39^ Whether or not other patients than these had experienced a more chronic MPN phase prior to their initial diagnosis of acute leukemia could not be ascertained from the review of the literature.

## Lymphoma

A number of reports have linked *PCM1-JAK2* fusions to the development of both Hodgkin’s disease (HD) and non-Hodgkin’s lymphoma. Remarkably, these cases can evolve over many years. In 1992, in the first reported case of *PCM1-JAK2* fusion, Davis described a patient with mycosis fungoides (MF) who was initially treated with radiation therapy.^55^ Over the course of 16 years this patient developed mixed cellularity CD30+ HD and eventually fatal cutaneous anaplastic large cell lymphoma. All 3 types of lymphoma demonstrated the same sequence of the T-cell receptor alpha chain.

Riedlinger also reported a patient with cutaneous T-cell lymphoma/mycosis fungoides.^56^ Twelve years later this patient developed what was diagnosed as HD. Eventually, the lymphoma transformed into large cell lymphoma. When lost to follow-up in year 17 the patient had stable disease. The cutaneous lymphoma, the HD, and the large cell lymphoma all possessed the *PCM1-JAK2* variant and the authors noted that what was initially diagnosed as HD may well have been an atypical form of T large cell anaplastic lymphoma.

Fernandez-Pol described 2 additional cases of MF with *PCM1-JAK2* variants out of 71 MF cases studied in detail at their institution.^53^ In year 6, the first patient’s T-cell clone was demonstrated in peripheral blood and in year 9 her disease transformed to CD 30+ T-cell large cell lymphoma. The second patient is alive 7 years after diagnosis with persistent mycosis fungoides. These authors also raise the question of whether or not the Davis and Riedlinger cases actually represent true HD. Both HD cases were CD30+ but also showed the T-cell clone of the cutaneous lymphomas, raising the possibility that while the histology may vary over time in such cases they may all be variants of the same large cell T-cell lymphoma rather than both HD and large cell lymphoma. This concept is supported by the recent report of 6 cases of a variety of *JAK2* rearrangements with peripheral T-cell lymphoma, one of which harbored the *PCM1-JAK2* fusion. These cases were all CD30+ ALK^-^ and the authors commented on the presence of Hodgkin-like features with Reed-Sternberg-like cells in all of them. No clinical follow-up data are available for the patient with the *PCM1-JAK2* variant.^54^

Additionally, in Schwaab’s recent report of cases of *PCM1-JAK2*-related MPN cases of HD and Burkitt’s lymphoma arose after successful HSCT for the myelodysplasia. The Burkitt lymphoma, which proved fatal, was noted to be positive for the fusion gene variant while the HD case was not.^27^

We recently reported a case in which a young woman presented with *PCM1-JAK2*-related MPN that transformed into T-cell ALL.^37^ She had a history of mixed cellularity HD-treated 13 years previously. However, we were unable to obtain adequate tissue from her HD to determine whether or not this was also caused by the fusion variant.

## Discussion

*PCM1-JAK2* fusion mutations are very uncommon and present with an overwhelmingly male predominance. The most frequent presentation is as an MPN, often, but not always, with eosinophilia. Erythrodysplasia can also be quite prominent and de novo leukemia of any lineage may be seen, including erythroleukemia. While cytogenetics usually will detect this variant there are patients who require specific probes, PCR and/or NGS.^16-19^

This variant usually produces aggressive disease. While there have been a number of good responses to ruxolitinib for MPN, the small number of patients reported precludes a definitive statement regarding its effect on survival. Similarly, HSCT has been successful for a number of patients but, again, analysis is limited by small numbers and insufficient follow-up. While the data presented here suggest that early HSCT may be more effective, this retrospective analysis lacks the scientific rigor of a randomized prospective trial and is not conclusive. Since only 2 patients have been treated with ruxolitinib for acute leukemia it is unclear if this drug has a role once acute leukemia has developed, though the report of a stable hematologic response in B-cell ALL is encouraging.^27,50^ Thus, HSCT or experimental therapy has been recommended to be considered early in the course of disease.^8,27^

Occasional patients presenting with MPN can do well for long periods of time either with ruxolitinib or even without treatment. It seems reasonable to consider initial treatment for MPN with symptomatic care such as hydroxyurea or interferon or a *JAK2* inhibitor if the patient is stable. However, there are no data to suggest that *JAK2* inhibitors are curative for this condition. HSCT is, for now, the only potentially curative treatment. All patients with *PCM1-JAK2* fusion mutation-related MPN should be evaluated at a transplantation center early in their course. It is important that biomarkers be developed to help guide the timing of transplantation.

The outlook is quite poor for patients presenting with or transforming into AML. These patients should receive induction therapy and undergo HSCT or experimental therapies. Whether the 2 transformed patients with ALL noted here who have done well with HSCT represent a leukemia subgroup with better prognosis remains to be seen with additional cases and follow-up. The favorable response of the one patient with ALL treated with ruxolitinib may be significant as well.

Since this variant affects a range of hematopoietic cell lines lymphomas can also be seen. As noted, there has been one case of Burkitt’s lymphoma.^27^ Of note, 5 patients have now been reported with cutaneous T-cell lymphoma/mycosis fungoides.^53-56^ All with follow-up data have survived at least 7 years, though 3 of the 4 have transformed into large cell lymphoma. There is not sufficient information to draw any conclusions regarding whether or not these patients behave differently from similar T-cell lymphomas that do not possess the *PCM1-JAK2* variant. Though 2 of these patients were initially thought to have HD, there is doubt as to whether they truly had both HD and large cell lymphoma or T large cell variants that looked similar to HD.^53-56^ Finally, we are unaware of any reports of *JAK2* inhibitor use for these lymphomas. It would be quite informative to evaluate their use in such cases.

## Conclusion

This rare condition may be increasingly detected with wider use of genomics. Ruxolitinib treatment can lead to hematologic or molecular improvement but further studies are needed to determine how best to utilize it. HSCT has demonstrated good results in patients, particularly in the absence of acute leukemia. At this point in time it remains the only potentially curative treatment though very small sample size, lack of random assignment to treatment and often short follow-up make comparisons between treatments underpowered and outcomes difficult to assess. Given the limitations of the data, at this point in time symptomatic care, ruxolitinib or experimental therapy can be considered for patients with stable MPN and may serve as a bridge to HSCT but the development of biomarkers to help determine timing of HSCT would be helpful. The outlook for patients presenting with acute leukemia is poor. These patients should be considered for aggressive treatment. Whether or not *JAK2* inhibitors are effective against acute leukemia or lymphoma caused by this fusion remains to be determined.

## Data Availability

The data underlying this article will be shared on reasonable request to the corresponding author.
